# Village doctors: a national telephone survey of Bangladesh’s lay medical practitioners

**DOI:** 10.1186/s12913-023-09972-w

**Published:** 2023-09-07

**Authors:** Olav Muurlink, Nazim Uzzaman, Rhonda J Boorman, Sarah Binte Kibria, Talitha Best, Andrew W. Taylor-Robinson

**Affiliations:** 1 CQUniversity, Brisbane, Australia; 2 University of Edinburgh, Edinburgh, UK; 3 Chittagong Medical College, Chittagong, Bangladesh; 4 College of Health Sciences, VinUniversity, Hanoi, 100000 Vietnam; 5 Center for Global Health, Perelman School of Medicine, University of Pennsylvania, Philadelphia, PA 19104 USA

**Keywords:** Traditional medical practitioners, Developing Countries, Medicine, traditional, Professional practice, Delivery of health care, Community pharmacy services, Fees, Medical, Complementary medicine, Medical education & training, Primary health care

## Abstract

**Background:**

Bangladesh outperforms its Least Developed Country (LDC) status on a range of health measures including life expectancy. Its frontline medical practitioners, however, are not formally trained medical professionals, but instead lightly-trained ‘village doctors’ able to prescribe modern pharmaceuticals. This current study represents the most complete national survey of these practitioners and their informal ‘clinics’.

**Methods:**

The study is based on a national Computer Assisted Telephone Interviewing (CATI) of 1,000 informal practitioners. Participants were sampled from all eight divisions and all 64 districts of Bangladesh, including 682 participants chosen from the purposively recruited Refresher Training program conducted by the International Centre for Diarrhoeal Disease Research, Bangladesh (ICDDR,B), supplemented with 318 additional participants recruited through snowball sampling.

**Primary and secondary outcome measures:**

In addition to demographics, village doctors were asked about the characteristics of their ‘clinics’ including their equipment, their training, income and referral practices.

**Results:**

Three quarters of the wholly male sample had not completed an undergraduate program, and none of the sample had received any bachelor-level university training in medicine. Medical training was confined to a range of short-course offerings. Village doctor ‘clinics’ are highly dependent on the sale of pharmaceuticals, with few charging a consultation fee. Income was not related to degree of short-course uptake but was related positively to degree of formal education. Finally, practitioners showed a strong tendency to refer patients to the professional medical care system.

**Conclusions:**

Bangladesh’s village doctor sector provides an important pathway to professional, trained medical care, and provides some level of care to those who cannot afford or otherwise access the nation’s established healthcare system. However, the degree to which relatively untrained paramedical practitioners are prescribing conventional medicines has concerning health implications.

## Background

Two thousand and twenty one marked the 50^th^ anniversary of Bangladesh’s independence, prompting scholars in a variety of disciplines to appraise the progress that the relatively young nation has made. A combination of governance and administrative delays including to the conduct of the national census, and the turmoil of Bangladesh’s early years means that baselines for the nation’s development are only approximate. In 1971, well over half the nation were estimated to be illiterate, and, with no longstanding tradition of recording births with any precision [1], even the population of the nation at inception can only be estimated. The health and economic metrics that do exist, however, suggest an emergent country in crisis: undernourishment impacted half the population; life expectancy was estimated at 40 years for men and 45 for women; and 15% of Bangladeshi children died within their first five years [2].

Today, life expectancy in Bangladesh exceeds that of India and Pakistan [3]. This is only part of what scholars have termed the ‘Bangladesh paradox’ [4], extraordinary progress on health indicators despite pervasive poverty. A combination of sustained reductions in birth rate (a reduction in family size of almost one child per family per decade) and mortality has been achieved on a limited budget. In the intervening five decades, Bangladesh’s health system has met a range of Millennium Development Goals, notably the reduction in the under-five mortality rate, containment of HIV infection, a sharp decrease in the prevalence of underweight children, a fall in both the infant mortality rate and maternal mortality ratio, improving coverage of vaccination and lowering the rate of communicable diseases, amongst others [5]. Bangladesh, which remains an LDC, is, to use the term of Yale political scientist Harry Blair, “punching above its weight” [6].

This success is difficult to explain simply by pointing to improvements in Bangladesh’s traditional medical infrastructure or its spending on health proportionate to GDP. At the birth of the nation, there were just eight medical college hospitals and 19 district hospitals; now there are 53 district hospitals alone, in addition to numerous private hospitals and clinics [7]. While the number of doctors has increased ahead of population growth, the number of nurses (for example) has not (World Bank Data). The nation’s success is also remarkable relative to its neighbours, with GDP per capita spend on health in Bangladesh at half that of India, and two thirds of that of Pakistan [7], both of which, as noted, have lower mean life expectancies. Improvements in Bangladesh’s national health outcomes have also outpaced conventional healthcare infrastructure and resource ratios [8].

Bangladesh’s strength in the implementation of cheap mass public health measures, in particular sanitation, clearly explains part of its unusual success [9]. Yet, the country is also unique in having a highly pluralistic workforce to address its health needs [10]. The informal health workforce dwarfs the number of medically-trained physicians, commonly known within Bangladesh as “MBBS doctors”, with collective estimates of traditional medical practitioners, homeopathic practitioners, drug shop attendants, and village doctors at 495,000, as opposed to 145,000 doctors, nurses, paramedics, and community health workers. The largest single category in the analysis of Ahmed et al. [10] was village doctors (unregulated, typically lacking formal medical training but practicing a form of allopathic medicine), also known as lay rural medical practitioners (RMPs) or *palli chikitshok,* approximated to be 185,000 [7]. The *palli chikishok* sector had early government backing in Bangladesh, with the support of the United States Agency for International Development [11] but that early push to provide a uniform single-year training that identifies this sector of ‘barefoot doctors’ practicing medicine based on allopathic principles of anatomy, physiology, pharmacology and nutrition, has long since ceased.

A probability sampling approach using the Bangladesh Bureau of Statistics’ (BBS) 2007 Sample Vital Registration System showed that there were around 12 village doctors per 10,000 population [12], strongly suggesting the national count of village doctors is still higher than the very imprecise published numbers. Since 2007 there has been no systematic attempt to reduce the freedom of operation of these unregulated medical practitioners. Thus, extrapolating this ratio to the likely 2023 population of approximately 170 million suggests that over 200,000 village doctors are now actively practicing in Bangladesh, although some methodologies based on local counts indicate that the number is lower [13].

Building an accurate portrait of this ‘army’ of amateur, unqualified or at least under-qualified, medical practitioners is therefore critical to understanding the healthcare capacity of the world’s eighth most populous nation. This is particularly important in understanding how health care is delivered to those of lower socioeconomic status, many of whom live in rural and regional Bangladesh where the shortage of MBBS doctors is acute. A partial impression of these village doctors can be gained from a large cluster of studies that examined Chakaria, in Cox's Bazar District in the Division of Chittagong (now officially known as Chattogram). Chakaria was chosen by the largest public health research facility in Bangladesh, the International Centre for Diarrhoeal Disease Research, Bangladesh (ICDDR,B), due to its profile as a “typical rural area in Bangladesh sharing socioeconomic and demographic characteristics of the low performing areas of rural Bangladesh” [14]. Mahmood et al. [15] sampled 1,000 randomly selected households in a locality that is neither the poorest nor the most remote in the country, finding that 44% of those surveyed had suffered illness in the previous 14 days, with 47% of those seeking treatment; of these almost two-thirds consulted village doctors, while just 14.2% consulted an MBBS doctor [15]. A relatively small qualitative study by Rahman et al. [16] was conducted in the same region as the research of Mahmood et al. [15]. This explored the village doctors’ perspective, finding a group lacking high levels of formal education, and a very heavy reliance on sales representatives of pharmaceutical companies for guidance [17]. The ICDDR,B’s exhaustive headcount of allopathic informal health practitioners found 328 to be working in the Chakaria region, in addition to substantial numbers of traditional and homeopathic health practitioners, religious healers, and traditional birth attendants [13]. That project provided a snapshot of the career pathways of village doctors, including training, as well as characteristics of their ‘clinics’, and approaches to treatment. However, the insight offered is now outdated, and is unlikely to be representative of the broader situation in Bangladesh. While there have been studies that focused on determining the knowledge of village doctors related to a single disease, for example [18], there is clearly a need for a greater understanding of village doctors’ breadth, currency and acquisition of knowledge [19].

This current study represents the most complete national survey conducted with the aim to investigate key characteristics of the largest sub-class of informal practitioners in Bangladesh’s health system, village doctors.

## Methods

### Study setting

Data collection was conducted by the International Centre for Diarrhoeal Disease Research, Bangladesh (ICDDR,B), the largest health research body in Bangladesh. Ethics approval (No. 0000021155) was obtained from the participating university, and separately from internal ethics review board at the ICDDR,B. The ICDDR,B is the largest and oldest public health research institution in Bangladesh, formed out of the SEATO Cholera Research Laboratory before the Liberation War, and maintains strong links with government and non-government health authorities at a district level.

### Participants and sampling

For this study, 1,000 RMPs were interviewed by telephone. Participants were sampled in two ways. Firstly, 1,000 of the 1,812 lay rural medical practitioners who participated in a Refresher Training program (RT group) run by ICDDR,B. Recruits to the RT program were purposefully recruited. For the purpose of this study, a random sample of 1,000 were drawn from this group of 1,812 and telephone contact was initiated. In total, contact was made with 682 RMPs and all agreed to participate (i.e. a 100%). Of the remaining RMPs, 318 were uncontactable after three attempts. A second wave of participants were recruited using a snowball technique, asking participants in the first wave for contact details of other practitioners working in their vicinity. Telephone contact was attempted with the referred 379 RMPs. Of these, 318 RMPs agreed to participate (response rate was 83.9%) with two declining to participate and 59 uncontactable after three attempts. Response rates in both waves were high due to the prestige attached to the ICDDR,B.

The resultant sample included village doctors from each of the eight divisions and all 64 districts of Bangladesh. The two most urban divisions of Bangladesh, Dhaka, and Chittagong BBS [20] are relatively underrepresented in this sample and the other six divisions oversampled, with the exception of Mymensingh (see Table 1). A key dimension in understanding Bangladeshi demographics from a health perspective is urbanisation, with urban residents more likely to have significantly fewer children, lower child and maternal mortality rates, higher income, higher literacy and educational attainment, and a higher age at marriage — all differences with significant health implications [21]. In the following, district (rather than divisional) level population density is used to explore for urban/rural effects. The distribution of density by district is not normal due to the presence of two districts in the Dhaka division, Dhaka district and Narayanganj district, which are respectively over 4 and 8 times the mean population density of the other 62 districts. We report results without transformation of data, with the exception of academic qualifications, when the skewed nature of the data impacted on the reported relationships.

**Table 1:** Sample source breakdown by division

**Division**	**Share of sample(%)**	**Share of population**^**a**^ (%)	
**Barisal**		8.4	5.7
**Chittagong**		12.3	20.0
**Dhaka**		19.2	25.0
**Khulna**		15.5	10.8
**Mymensingh**		7.5	7.8
**Rajshahi**		14.6	12.7
**Rangpur**		12.9	10.9
**Sylhet**		9.6	6.7

^a^BangladeshBureau of Statistics, Population and Housing Census

### Study design

The survey was conducted by telephone, and coded into a Computer Assisted Telephone Interviewing (CATI) system. Telephone interviews were undertaken by trained research assistants working with the ICDDR,B. The survey was conducted in 2019 prior to lockdowns caused by the COVID-19 pandemic. The interview team took informed verbal consent from participants and recorded the interviews without co-recording identity. Research assistants transferred the data from the audio recordings into a database.

### Survey description

Respondents were requested to provide their age and gender. They were asked *where* they operated (own drug shop, hereafter ‘pharmacy’, another’s ‘pharmacy’, their residence, a separate chamber, or otherwise), the *duration* of their practice (to the nearest year), current patient load and patient type (infant, child under 12 years of age, and the gender ratio of patients older than 12), their referral *frequency* (to a community clinic, *upazilla* (local district) health clinic, district hospital, government medical college hospital, private hospital or clinic, or to a specialist hospital), their monthly net income, and their equipment (such as stethoscope, blood pressure monitor, thermometer, diagnostic penlight, scales, suture kit, glucometer and nebulizer). They were also asked for details of their relevant qualifications and training.

### Statistical analysis

Descriptive statistics included frequencies and percentage analyses, as well as central tendency analysis using means and standard deviations. Differences between urban and rural groups were assessed by *t*-test. For categorical information, non-parametric *chi*-square analysis was used. All data were managed through SPSS and no data imputation was used.

Income raw data were positively skewed with a small proportion of village doctors reporting very large incomes. Common logarithm was performed, which made variables involving income distribution less skewed [22]. Inferential statistical analyses involving income were conducted using both raw and transformed data. In all analyses, the outcomes were comparable and therefore only the raw data results are reported. A preliminary analysis of differences between the Refresher Training (RT) group and the balance of participants was conducted with differences between the groups at times statistically significant, but with a small effect size. For example, there was a significant difference in age of the RT group (40.99 years) and non-RT group (42.8 years) with a small effect size (Cohen’s d=.18) and academic qualifications were significant, again with a small effect size (Cohen’s d=.14). Unsurprisingly, there was a significant difference (*p* = .05) in number of professional qualifications of the RT group (2.13) and non-RT group (1.78) with a small/moderate effect size (Cohen’s d = .43).

## Results

### Demographic characteristics of village doctors

The sample of 1,000 offers a broad description of RMPs across Bangladesh’s diverse 64 divisions. Significantly, all participants were male even though this was not a requirement for participation. Key demographic characteristics are presented in Table 2.

**Table 2:** Key sample demographics of village doctors (*n*=1,000)

Age	
Median (IQR) years	40 (34-48)
Mean (SD)	41.6 (10.3)
Years of practice	
Median (IQR) years	16 (10-23)
Mean (SD)	17.6 (10.1)
Location population density	
Median (IQR) people per square km in thousands	1117 (923, 1332)
Mean (SD) people per square km in thousands	1575.3 (1957.0)
Highest Academic qualifications, number (%)	
Did not complete SSC	10 (1)
SSC completed	309 (31)
HSCcompleted	430 (43)
Bachelor	188 (19)
Master	58 (6)
Total monthly income	
Median (IQR) Taka	20,000 (13-30K)
Mean (SD) Taka	26,086 (25,730)
Income per patient	
Median (IQR) Taka	234 (146-385)
Mean (SD) Taka	311.2 (294.0)
Number of patients per month	
Median (IQR)	87 (52-128)
Mean (SD)	101.2 (70.1)
Professional qualifications, number (%)	
None	13 (1.3)
RMP	770 (77)
LMA	518 (52)
PC	262 (26)
MAT	50 (5)
Other	421 (42)
Total number of professional qualifications	
Median (IQR)	2.0 (1-3)
Mean (SD)	2.0 (0.8)
Min - Max	0-4
Ratio of referrals per patient	
Median (IQR)	0.29 (0.15-0.58)
Mean (SD)	0.48 (0.57)
Practice site, number (%)	
'Pharmacy', own	798 (80)
'Pharmacy', other	70 (7)
Own house	37 (4)
Separate chamber	79 (8)
Other	229 (23)
Equipment, number (%)	
Stethoscope	971 (97)
BP Machine	969 (97)
*wr* Machine	827 (83)
Torch	933 (93)
Thermometer	969 (97)
Nebulizer	662 (66)
Glucometer	771 (77)
Stitching instruments	804 (80)
Total number of equipment	
Median (IQR)	7.0 (6-8)
Mean (SD)	7.0 (1.2)

The mean age of RMPs was 41.5 years of age and they typically worked out of their own ‘pharmacy’.

### Educational attainment

Village doctor education level (years of school attendance *M*=13.39, *SD*=2.40) was significantly higher (*p*<.001) than that of the population (which in 2019 was 6.06 years [23]). One-third had a secondary school certificate (SSC — 10 years of schooling) or less, while 43% had a higher school certificate (HSC; completion of 12 years at school). This figure of 43% is above the latest *urban* mean (35%), let alone lower rural rates [24]. While education level was above population norms, three quarters of participants did not have education past high school.

Almost one-fifth (19%) of participants had completed a Bachelors’ degree and 6% had a Masters’ qualification, but it is important to note that these qualifications are not medical qualifications. Most had completed at least a single professional training course, regardless of whether they were in the RT group or the snowball group.

Table 2 shows that the most common qualification was RMP/PC (Rural Medical Practitioner/*Polli chikitsok*), a course of three months’ duration covering basic anatomy and physiology, pharmacology, microbiology and pathology as well as first aid and some professional elements of the practice of medicine. Minimum qualification for entry to an RMP course is a secondary school certificate and having some experience of medical science [25]. The Local Medical Assistant and Family Planning (LMAFP) course is a longer eight-month version of the RMP, covering an additional family planning component [25].

The least frequently mentioned was the Medical Assistant Training (MAT) course, which is a relatively comprehensive three-year course with an additional one-year internship [26]. There were no significant differences in academic attainment or professional qualifications/training when the transformed density variable was used.

### Profile of clinical resources

The majority of village doctor ‘clinics’ are co-located in their own ‘pharmacy’ (80%).

Participants were asked about their access to eight pieces of equipment used for routine medical examination. Almost half of participants (*n* = 481) reported having all eight items. Nearly 80% had five or more of this basic ‘tool kit’. Of the set, the most commonly available were stethoscope, thermometer, blood pressure monitor, and torch. The least common item was a nebulizer. A significant positive correlation (*p* < .001) was observed between number of items of equipment and level of training, with a small effect size (*r* = .13). Also, there was a small but significant effect (*p* < .001, *r* = .20) for the relationship between number of pieces of equipment and total monthly income.

### Patient case load

RMPs reported to treat on average 100 patients per month. Patient load increased significantly with increasing years of experience spent in practice (*r* = .12, *n* = 993, *p* < .001) but not with age (*r* = .06, *n* = 996, *p* = .08). As the patient load increased, not surprisingly monthly income increased (*r* = .32, *n* = 978, *p* < .001), but the income per patient decreased (*r* = -.27, *n* = 978, *p* < .001). The relationship between population density and total monthly patients was not significant.

### Case load through referrals

RMPs reported the frequency of referrals that they made to other service providers. This ranged from 0 to 415 referrals per month. Nearly all (99.4%) referred at least one patient to one of the target medical facilities and 90% completed at least seven referrals each month.

A variable representing referrals per patient was calculated by dividing the number of referrals by the number of patients that each respondent reported. Expressed as a ratio, this variable reflects the likelihood of a village doctor to refer patients for standard medical examinations or treatment. Broadly speaking, a ratio of 0.5 indicates that half the patients were referred, and 1.0 indicates all patients were referred. It is possible for a village doctor to refer an individual patient to more than one medical facility, which results in some case ratios exceeding 1.0. In fact, ratios ranged from 0.0 to 6.2. The presence of extreme ratios well above 1.0 may be a consequence of participants being asked to estimate multiple factors involving patient referrals to a range of target institutions, which were then combined to generate the final count. The authors consider it misleading to remove the extreme ratios from the dataset because the distribution of the variables was normal. While figures may be inflated, they are likely to be inflated consistently. Therefore, all analyses were conducted using the full range of ratio data.

On average, the unadjusted figures suggest that almost half of all patients (*M* =.48, *SD* = 0.57) were referred for further examinations or treatment. However, the median was 25% of patients being subject to referral.

Simple correlations showed that the ratio of referrals to patients was slightly negatively associated with both age of the practitioner (*r* = -.07, *n* = 975, *p* = .024) and with years in practice (*r* = -.09, *n* = 973, *p* = .005). That is, higher rates of referral per patient were statistically associated with lower age and less experience of the practitioner. There was no significant association with education (*r* = -.00, *n* = 972, *p* = .94) or professional qualifications (*r* = -.05, *n* = 975, *p* = .123). There was a positive correlation between referral ratio and a practitioner’s income per patient, with increases in ratio associated with increases in income per patient (*r* =.11, *n* = 9957, *p* < .001). Urban doctors were significantly more likely to refer than were rural doctors. Once again, no relationship between population density and referral patterns emerged.

### Income

RMPs in our sample earned a mean income of 26,086 taka per month, more than double the currently applicable minimum wage of 12,016 taka [27] (roughly equivalent to $USD120). RMPs reported on monthly income from two sources: patient consultancy; and pharmaceutical sales. Most (79%) did not receive income from consultancy, with only 67 practitioners (7%) earning income exclusively from consultancy, that is not supplemented by the sale of pharmaceuticals (see Table 3). For the whole sample, mean ‘pharmacy’ income (*M* = 21,628, *SD* = 24,123) was almost five times greater than consultancy income (*M* = 4457, *SD* = 11,402). A significant relationship between population density and income emerged with a small effect size (*r* = 0.12, *n* = 979, *p* < .001).

**Table 3:** Breakdown of income by income source

**Clinic monthly income source breakdown** (%)		
	Consultancy	Pharmacy
0 taka	79	7
500-4,999 taka	1	3
5,000-9,999taka	2	11
10,000-19,999taka	9	32
20,000-29,999taka	4	21
30,000+ taka	5	24

Correlation analyses were conducted to investigate the influence of qualifications held by the village doctor on income(total). There was a significant positive correlation between monthly income and number of qualifications (*r* = .11, *n* = 979, *p* < .001), indicating that income tended to increase in line with number of qualifications. Similarly, there was a positive correlation between monthly ‘pharmacy’ sales income (which forms part of total monthly income) and number of qualifications (*r* = .10, *n* = 979, *p* = .002). There was no significant difference in total income per patient when compared across the number of qualifications. The relationship between *level* of education and monthly income was significant (*r* = .09, *n* = 976, *p* = .006), but the effect size was small. Hence, a one-way ANOVA —which compares means (monthly income) at each level of education — does not reach significance; *F* [5] = 1.39, *p* = .18 (*p* = .224). Level of education was not associated with number of qualifications; (X^2^ (8, *n* = 995) = 2.69, *p* = .95).

## Discussion and conclusion

Bangladesh suffers from a “shortage of, and geographic maldistribution” of its professional medical staff [28]. While 10,000 new MBBS graduates qualified to practice medicine emerge from Bangladesh’s medical schools each year [29], this cohort often resists assignments to rural postings. Where rural assignments are accepted, absenteeism is rife [17, 30, 31]. To fill this gap in healthcare, there exists a large, highly pluralistic workforce that includes a substantial number of amateur (non-medically trained) medical providers [29, 32]. This study provides a snapshot portrait of the dominant informal subsector, the village doctor, a role that not just responds to the healthcare needs of the underserviced, but also populations that cannot afford standard medical services. It is clear from the findings that there is significant diversity within this sector, with a range of qualifications standing in contrast with the early *palli chikitshok* program. What constitutes a ‘village’ in Bangladeshi terms may not match dimensions elsewhere; this survey included operators in the relatively dense divisions, and anecdotally, the village doctors operate wherever geography or patient ability to pay qualified medical doctors places a ‘natural’ limit on the willingness of qualified practitioners to set up practice.

In contrast to formally trained medical practitioners, village doctors are in plentiful supply and are culturally and socially embedded in rural communities. These amateur medical operators run very small ‘clinics’ —seeing only around 100 patients a month—and earn an income that, while above the minimum wage, falls well below a recent estimate of the median salary of entry and mid-level banking employees in Bangladesh, for example [33]. While 100 patients a month may be a low patient load relative to that seen by conventionally-trained general practitioners, considering the sheer number of village doctors (around 200,000) at work in Bangladesh, it still indicates that each citizen sees a village doctor more than once a year.

This study has limitations in sampling, insofar as the core of the sample are those who have received some training from the ICDDR,B and the self-report nature of the data. The ICDDR,B is a nationally recognised healthcare, research and training ‘brand’ which may have impacted the representativeness of the sample. It is worth considering, for example, that data collected by a prestigious Dhaka-based health institute may systematically and significantly under-report patient load, over-claim qualifications, or over-report referrals in deference to the reputation of the ICDDR,B. If the data systematically presents an overly cautious picture of the practice of village doctors (who may be aware of the fuzzy legal status of their work), that is cause for additional concern. The study also does not address the question of the quality of the training received or the retention of critical knowledge delivered to participants during training.

Perhaps the key finding of this investigation is that village doctors are lacking not just in terms of substantial conventional medical training, but also for a formal education in general. Thus, 75% have at most completed high school, with the balance of their training coming from short course ‘qualifications’. Earlier research shows that degree-qualified village doctors’ qualifications are in non-medical fields such as Islamic Studies or Business [34]. In and of itself, this shows an awareness of and respect for a need to be qualified in an area of practice. However, the level of training is well below that of a qualified medical practitioner.

One of the extraordinary achievements of Bangladesh has been the development and growth of a domestic pharmaceutical industry, now meeting almost the entirety of domestic demand [3]. However, paired with an unregulated pharmaceutical marketplace in the country, the industry is able to supply ‘pharmacies’ which are collocated with village doctor ‘clinics’, and these pharmacies in return are able to respond directly to consumer demand, limited only by consumer purchasing capacity [35]; Rasu and colleagues [36] refer to the “prevalent cultural pressure from patients to prescribe something”. Equally concerningly, with a low patient load the village doctors operating these pharmacies are highly dependent on income derived directly from the sale of pharmaceuticals, prescribing medicines that they retail themselves. Over three quarters of the sample (78%) derived *no* income from consultation fees whatsoever and were entirely dependent on the sale of pharmaceuticals for their income. Previous analysis shows that the majority of medicines that they dispense (not surveyed directly in this study) are conventional, regulatory authority-approved medicines, ranging from painkillers to antibiotics [37]. Isolated from formal training or support, the practitioners are vulnerable to influence from representatives of pharmaceutical companies who act as a primary source of medical knowledge [16].

This study does however illustrate a curious paradox: an army of amateur doctors working outside the prevalent laws restricting the prescription of pharmaceuticals [36] working in the shadows of—and to some degree coordinating with—their conventionally-trained counterparts. Village doctors in fact show considerable willingness to defer to trained medical professionals, running ‘clinics’ equipped with the basic accoutrements of modern medicine (the stethoscope, blood pressure monitor and modern pharmaceuticals) and perhaps, with the limited training qualifications that they report, this is a wise community position to hold. Almost the entire sample referred a patient at least once a month, with the median proportion of patients subject to referral being around 25%. This relationship may not damage the business of the village doctor, as it seems to reflect an underlying “knowingness” that this is the socially and culturally accepted role that the village doctor fulfils. This is borne out by the fact that the proportion of patients referred on were not associated with a reduced income on the part of practitioners. Village doctors appear to see value in integrating, to some extent, with mainstream health services that are accessed by referral. Hence, they are acting in concert with qualified medical practitioners. Previous studies have indicated that RMPs gain medical knowledge from their connection with mainstream medical providers, just as they do from networking with pharmaceutical representatives. For the *palli chikitshok*, the relationship with professional medical providers potentially offers referred status, as well as a safety net in the event of the occurrence of serious cases. A propensity to refer may be considered as a form of insurance for the village doctor, while ‘success’ with minor cases, combined with the passing on of serious cases, can ensure continued reputational status in their village.

Referral frequency conceivably is related to the availability of accessible medical facilities such as clinics or hospitals, but the data suggests otherwise—the practice of referral was not related to population density. There is no substantial research on attitudes of medical professionals in Bangladesh *towards* village doctors, but referral patterns do suggest a positive attitude towards medical professionals on behalf of the practitioners doing the referrals. Referral rates by village doctors fall within the range of frontline medical practitioners in the UK and US for example—a large cohort study showing that in the UK the rate is around 14%, while in the US, rates are above 30% [38].

Perhaps unsurprisingly, there appears to be no appetite at a national level to eliminate village doctors from the Bangladeshi healthcare system, despite their operating outside the letter of the law. This ‘army’ of amateurs delivers almost exclusively allopathic medicine in niche geographical and economic sectors where the conventional health care system fails to reach. It is closely connected to the mainstream healthcare system, and future studies are required to map how citizens connect with the mainstream, and the role that village doctors play in that system. The study shows that village doctors are almost exclusively male, with this study locating only male practitioners. The gender imbalance has obvious implications for women’s health in a patriarchal society, which remain relatively unexplored in the literature.

The picture that emerges from this study is of an informal healthcare sector that mimics many of the characteristics of the formal healthcare system. The degree to which village doctors engage in the sale of pharmaceuticals, which are largely allopathic medicines, their willingness to engage in training, albeit brief, that fits within the allopathic paradigm, and the tendency to refer cases into the nation’s conventional healthcare system, speak to a system that is not actively working *against* the current of mainstream medicine, but in its slipstream. The distinction between the *prescription* and *selling* of pharmaceuticals remains blurred in Bangladesh. Progress in improving the safety of the sector will almost certainly require a greater willingness at a regulatory level to acknowledge not just that the sector exists but *why* it exists. Bangladesh’s population is mainly rural-based, and like rural populations in the developing world (e.g. [39] timely access to healthcare remains a problem; Bangladesh’s willingness to allow breaches of regulation to proliferate no doubt partially addresses this problem, but the side-effects of the ‘solution’ remain troubling.

## Data Availability

All data underlying the findings in this paper are fully available without restriction upon request, subject to adherence to ICDDR,B’s data sharing policy (http://www.icddrb.org/policies). Requests for data can be sent to nazim.uzzaman@icddrb.org, co-lead investigator.

## Abbreviations

ICDDR,B: International Centre for Diarrhoeal Disease Research, Bangladesh
CATI: Computer Assisted Telephone Interviewing system
